# Harnessing Metabolic Regulation to Increase Hfq-Dependent Antibiotic Susceptibility in *Pseudomonas aeruginosa*

**DOI:** 10.3389/fmicb.2018.02709

**Published:** 2018-11-09

**Authors:** Petra Pusic, Elisabeth Sonnleitner, Beatrice Krennmayr, Dorothea A. Heitzinger, Michael T. Wolfinger, Armin Resch, Udo Bläsi

**Affiliations:** ^1^Max F. Perutz Laboratories, Department of Microbiology, Immunobiology and Genetics, Vienna Biocenter, University of Vienna, Vienna, Austria; ^2^Department of Theoretical Chemistry, University of Vienna, Vienna, Austria

**Keywords:** *Pseudomonas aeruginosa*, Hfq, antibiotic susceptibility, c-di-GMP, membrane potential

## Abstract

The opportunistic human pathogen *Pseudomonas aeruginosa* is responsible for ~ 10% of hospital-acquired infections worldwide. It is notorious for its high level resistance toward many antibiotics, and the number of multi-drug resistant clinical isolates is steadily increasing. A better understanding of the molecular mechanisms underlying drug resistance is crucial for the development of novel antimicrobials and alternative strategies such as enhanced sensitization of bacteria to antibiotics in use. In *P. aeruginosa* several uptake channels for amino-acids and carbon sources can serve simultaneously as entry ports for antibiotics. The respective genes are often controlled by carbon catabolite repression (CCR). We have recently shown that Hfq in concert with Crc acts as a translational repressor during CCR. This function is counteracted by the regulatory RNA CrcZ, which functions as a decoy to abrogate Hfq-mediated translational repression of catabolic genes. Here, we report an increased susceptibility of *P. aeruginosa hfq* deletion strains to different classes of antibiotics. Transcriptome analyses indicated that Hfq impacts on different mechanisms known to be involved in antibiotic susceptibility, viz import and efflux, energy metabolism, cell wall and LPS composition as well as on the c-di-GMP levels. Furthermore, we show that sequestration of Hfq by CrcZ, which was over-produced or induced by non-preferred carbon-sources, enhances the sensitivity toward antibiotics. Thus, controlled synthesis of CrcZ could provide a means to (re)sensitize *P. aeruginosa* to different classes of antibiotics.

## Introduction

*P. aeruginosa* is a Gram-negative opportunistic pathogen that is associated with a broad spectrum of acute and chronic infections (Kerr and Snelling, 2009). *P. aeruginosa* can be devastating for immunocompromised individuals or patients with cystic fibrosis, leading to high morbidity and mortality (Williams et al., 2010). A major burden to eradicate *P. aeruginosa* infections is its high intrinsic resistance against several classes of antibiotics (Poole, 2011) as well as its ability to form biofilms. Biofilms are recalcitrant to antibiotic treatment due to restricted penetration and their altered physiology (Ciofu et al., 2015). One of the key signals for the transition from mobile to sessile lifestyle is the signaling molecule cyclic-3′,5′-diguanylic acid (c-di-GMP) (Valentini and Filloux, 2016). High c-di-GMP levels correlate with biofilm formation and low c-di-GMP levels with planktonic growth (Simm et al., 2004; Basu Roy and Sauer, 2014). The cellular content of c-di-GMP is established by the interplay between diguanylate cyclases (DGC) and phosphodiesterases (PDE) that are responsible for the synthesis and degradation of c-di-GMP, respectively (Valentini and Filloux, 2016). The c-di-GMP levels are sensed by c-di-GMP binding receptors/effectors (Valentini and Filloux, 2016; Moradali et al., 2017), which then activate exopolysaccharide production (Psl, Pel, alginate), cell aggregation and biofilm formation, and interfere with the ATPase activity of the master regulator of flagellar biosynthesis FleQ (Su et al., 2015). Moreover, elevated levels of c-di-GMP can interfere with antibiotic resistance in a manner unrelated to biofilm growth (Hoffman et al., 2005; Gupta et al., 2013, 2014; Nicastro et al., 2014). In addition to the c-di-GMP signaling pathway, the Gac/Rsm network also regulates the switch between planktonic and biofilm lifestyle (Mikkelsen et al., 2011). The Rsm system in *P. aeruginosa* consists of two RNA binding proteins (RsmA and RsmN) and four regulatory RNAs (RsmY, RsmZ, RsmW, RsmV) that function by sequestering RsmA and RsmN from mRNA targets (Pessi et al., 2001; Heurlier et al., 2004; Kay et al., 2006; Marden et al., 2013; Morris et al., 2013; Miller et al., 2016; Janssen et al., 2018). The regulatory RNAs are transcribed under different growth conditions and at least two of them, RsmZ and RsmY, are under the control of the two component system GacA/S (Kay et al., 2006; Miller et al., 2016; Janssen et al., 2018). Moreover, the levels of RsmZ are dependent on RNase G (CafA), the synthesis of which is controlled by the two component system BfiSR in a biofilm specific manner (Petrova and Sauer, 2010). There are some indications that c-di-GMP signaling and Gac/Rsm mediated regulation are interlinked (Frangipani et al., 2014; Moscoso et al., 2014). Most likely, RsmA and RsmN affect the synthesis of DGCs, like SiaD, RoeA and SadC at the post-transcriptional level (Moscoso et al., 2014; Romero et al., 2018).

Sub-populations of *P. aeruginosa* biofilms respond differently to antibiotic treatment due to their altered metabolic activity (Pamp et al., 2008). Similarly, different environmental cues impact on antimicrobial susceptibility (Martínez and Rojo, 2011; Poole, 2012), which has been attributed to regulators such as Crc (Linares et al., 2010), CbrA (Yeung et al., 2011) or PhoPQ (Schurek et al., 2009). Moreover, small regulatory RNAs (sRNA), which commonly act in response to stressors, can act as post-transcriptional regulators of genes involved in antibiotic susceptibility (Dersch et al., 2017). For example, the *P. aeruginosa* sRNAs Sr0161 and ErsA control meropenem resistance by binding to the 5′ UTR of *oprD* (Zhang et al., 2017), encoding an uptake channel for carbapenems. The *P. aeruginosa* sRNA Sr006 activates the expression of *pagL*, encoding an enzyme responsible for deacylation of lipid A, which results in polymyxin resistance (Zhang et al., 2017).

In *P. aeruginosa*, the global regulator Hfq is involved in sRNA-mediated riboregulation as well as carbon catabolite repression (CCR) (Sonnleitner and Bläsi, 2014; Ferrara et al., 2015; Sonnleitner et al., 2017; Zhang et al., 2017). The function of Hfq in riboregulation in Gram-negative bacteria has been well established. It stabilizes sRNAs and facilitates their annealing with target mRNAs (Santiago-Frangos and Woodson, 2018). In *P. aeruginosa*, Hfq was shown to bind directly to catabolic mRNAs to block translation during CCR (Sonnleitner and Bläsi, 2014). Hereby, the catabolite repression protein Crc acts as a co-repressor by stabilizing the RNA-Hfq complex (Sonnleitner et al., 2018). The response to different C-sources is mediated through different levels of the regulatory RNA CrcZ (Sonnleitner et al., 2009; Valentini et al., 2014). CrcZ can sequester Hfq when CCR is relieved (Sonnleitner and Bläsi, 2014; Sonnleitner et al., 2018). CrcZ expression is under control of the alternative sigma factor RpoN and the two component system CbrA/B (Sonnleitner et al., 2009; Abdou et al., 2011). The signal responsible for CbrA activation remains unknown, but it is thought to be related to the internal energy status of the cell (Valentini et al., 2014). Furthermore, as CrcZ acts as a decoy for Hfq, CrcZ does not only interfere with direct translational repression by Hfq-Crc (Sonnleitner and Bläsi, 2014) but also with Hfq-mediated riboregulation by sRNAs (Sonnleitner et al., 2017).

There is limited knowledge on the impact of Hfq on antibiotic susceptibility in bacteria (Yamada et al., 2010; Kim et al., 2015). The increased sensitivity of an *E. coli hfq* mutant strain toward several toxic compounds coincided with reduced quantities of ArcB, which is a component of the ArcAB-TolC efflux pump. Further studies indicated that Hfq positively regulates *arcB* at the post-transcriptional level (Yamada et al., 2010). As mentioned above, there are some examples in which functions involved in antibiotic susceptibility are directly or indirectly regulated by Hfq-mediated riboregulation via sRNAs (Dersch et al., 2017; Zhang et al., 2017). Here, we studied the susceptibility of *P. aeruginosa* PAO1 and PA14 *hfq* deletion strains toward several classes of clinically relevant antibiotics. In general, the *hfq* deletion resulted in an increased sensitivity toward the antibiotics. A RNA_Seq_ based transcriptome analysis of PA14Δ*hfq* indicated that Hfq impacts on several antibiotic resistance determinants. Moreover, the *hfq* deletion strains were impaired in energy metabolism and have reduced c-di-GMP levels. Furthermore, we show that cross-regulation by the regulatory RNA CrcZ, which titrates Hfq, renders *P. aeruginosa* wild-type strains more susceptible to antibiotics. Induced synthesis of CrcZ in complex synthetic cystic fibrosis medium reduced the minimal inhibitory concentration (MIC) toward gentamicin and cefepime.

## Materials and methods

### Bacterial strains, plasmids and growth conditions

The *P. aeruginosa* strains PAO1 (Holloway et al., 1979), PAO1Δ*hfq* (Sonnleitner et al., 2017), PA14 (Liberati et al., 2006), PA14Δ*hfq* (Wurtzel et al., 2012), and the plasmids pMMB67HE (vector control; Fürste et al., 1986) and pMMB*crcZ* (P_*tac*_ driven constitutive expression of *crcZ*; Sonnleitner and Bläsi, 2014) have been described. As indicated in the text, the cultures were grown at 37°C in either synthetic cystic fibrosis medium (SCFM) (Palmer et al., 2007) containing 100 μM FeSO_4_ (Tata et al., 2016), Lysogeny-broth (LB) (Sambrook et al., 1989) or Basal-Salt medium (BSM) supplemented with the indicated carbon sources (Sonnleitner et al., 2009).

### Construction of plasmid pTC*glpT*

To construct a transcriptional gene fusion between *glpT* and *lacZ*, a 468-bp fragment containing the *glpT* promoter region (nt −495 to −27 with regard to the A (+1) of the start codon) was amplified by PCR using the oligonucleotides D121 (5′-TTT TTG AAT TCC CTT CGC TGC CGG CCA ACC-3′) and E121 (5′-TTT TTC TGC AGG CCT TTT TAC GCG GTT GC-3′) and chromosomal DNA of PA14 as template. The PCR fragment was cleaved with *Eco*RI and *Pst*I, and then ligated into the corresponding sites of plasmid pME6016 (Schnider-Keel et al., 2000).

### Effect of gentamicin on PA14 and PA14Δ*hfq* grown to early stationary phase

Overnight cultures of PA14 and PA14Δ*hfq* were inoculated in SCFM at an initial OD_600_ of 0.05, and the cultures were further grown aerobically to an OD_600_ of 1.8–2.0. Then, 180 μl aliquots were transferred to 96-flat-bottom-well microtiter plates (Thermo Scientific) and 20 μl of SCFM containing serial dilutions of gentamicin (0.5–4 mg/L) were added. Static growth was monitored at 37°C for 18 h in a SynergyH1 microplate reader (Biotek). From these initial studies it became obvious that a gentamicin concentration of 2 mg/L resulted in a stronger growth retardation of PA14Δ*hfq* when compared with PA14 (not shown). Therefore, the growth experiment was repeated in the presence of gentamicin at a final concentration of 2 mg/L. Samples were withdrawn at an OD_600_ of 1.8–2.0 (initial inoculum) and after 18 h of incubation in the presence of 2 mg/L gentamicin. The samples were serially diluted and plated on LB agar plates. The plates were incubated overnight aerobically at 37°C to determine the CFU. All experiments were performed in duplicates and the results are presented as mean ± standard deviation.

### Determination of the minimal inhibitory concentration (MIC) by evaluator strips

Bacterial cultures were grown in the media as indicated in the text to an OD_600_ of 1.8–2.0. Then, either 200 μl of cultures (high inoculum, corresponding to 4 × 10^9^ CFU/mL) or 100 μl of 1/1,000 dilution of the respective cultures (low inoculum, corresponding to 2 × 10^6^ CFU/mL) were plated on agar plates containing the respective media. MIC Evaluator strips were applied with the following antibiotic concentrations: cefepime, 0.016–256 mg/L (bioMérieux); ciprofloxacin, 0.002–32 mg/L (Oxoid); colistin, 0.016–256 mg/L (bioMérieux); fosfomycin, 0.064–1,024 mg/L (bioMérieux); gentamicin, 0.015–256 mg/L (Oxoid); imipenem, 0.002–32 mg/L (Oxoid); tetracycline, 0.015–256 mg/L (Oxoid). The plates were incubated at 37°C and the MICs were determined by analyzing the growth inhibition zones. The MICs correspond to the lowest concentration of antibiotics that impeded growth.

### Agar disc diffusion

PA14 and PAO1 were grown in BSM supplemented with either 40 mM succinate or oxaloacetate to an OD_600_ of 1.8–2.0. Then 200 μl of cultures were plated on agar plates containing the respective media and filter discs were applied on top with the following antibiotic concentrations: cefepime (Sigma), 60 μg; colistin (Oxoid), 25 μg; ciprofloxacin (Oxoid), 5 μg; gentamicin (Roth), 120 μg. The filter discs for colistin and ciprofloxacin were purchased commercially (Oxoid), the other discs were self-made. The plates were incubated at 37°C. The diameter of the growth inhibition zones was measured and normalized to that obtained on BSM-succinate, which was set to 100%.

### MIC determination by microdilution in glass tubes

PA14, PAO1 and the PA14 and PAO1 strains, harboring either plasmid pMMB67HE or plasmid pMMB*crcZ*, were grown in SCFM medium to an OD_600_ of 1.0. Then, 1 ml of culture was mixed with 1 ml of SCFM medium containing serial dilutions of gentamicin (concentration 4 to 48 mg/L) or cefepime (concentration 40 to 5,000 mg/L) in the presence or absence of 40 mM oxaloacetate. The cultures were shaken at 37°C for 20 h and then the OD_600_ was measured and pictures were taken. The MICs correspond to the lowest concentration of antibiotics that impeded growth.

### Northern blot analysis

Total RNA of PA14, PAO1 and of PA14 and PAO1 harboring either plasmid pMMB67HE or plasmid pMMB*crcZ* (grown for 20 h at 37°C in SCFM in the presence or absence of 40 mM oxaloacetate) was purified with the TRIZol reagent (Ambion) according to the manufacturer's instructions. The steady state levels of CrcZ and 5S rRNA (loading control) were determined by Northern-blotting using 4 μg of total RNA. The RNA samples were denatured for 5 min at 65°C in loading buffer containing 50% formamide, separated on a 8% polyacrylamide/8 M urea gel, and then transferred to a nylon membrane by electroblotting. The RNAs were cross-linked to the membrane by exposure to UV light. The membranes were hybridized with gene-specific ^32^P-end-labeled oligonucleotides [CrcZ: K3 (5′-GCT GGA GTC GTT ACG TGT TG-3′); 5S rRNA: I26 (5′-CCC CAC ACT ACC ATC GGC GAT GCG TCG-3′)]. The hybridization signals were visualized using a PhosphorImager (Molecular Dynamics). The normalization was performed with ImageQuant TL Toolbox v8.1.

### RNA_seq_

Total RNA was prepared from two biological replicates of PA14 and PA14Δ*hfq* after growth in SCFM to an OD_600_ of 2.0. Total RNA was isolated using the TRIzol reagent (Ambion) according to the manufacturer's instructions. The samples were DNase I treated, followed by phenol-chloroform-isoamyl alcohol extraction and ethanol precipitation. The Ribo-Zero rRNA Removal Kit (Epicenter Biotechnologies) was used to deplete rRNA from total RNA samples. Libraries were constructed using the NEBNext1Ultra™ Directional RNA Library Preparation Kit from Illumina. 100 bp single end sequence reads were generated using the Illumina HiSeq 2000 platform at the Vienna Biocenter Campus Science Support Facility (https://www.vbcf.ac.at/facilities/next-generation-sequencing/). Quality control assessment, sequencing adapter removal and mapping of the samples against the PA14 reference genome (NCBI accession number NC_008463.1) were performed as described previously (Tata et al., 2016). Reads mapping to regions annotated as either rRNA or tRNA were discarded from the data. The mapped sequencing data were prepared for visualization using the ViennaNGS tool box and visualized with the UCSC Genome Browser (Wolfinger et al., 2015). Reads per genes were counted using BEDTools (Quinlan and Hall, 2010) and the Refseq annotation of *P. aeruginosa* (NC_008463.1). Differential gene expression analysis was performed with DESeq (version 1) (Anders and Huber, 2010). The levels of transcripts with a fold change greater than ± 4 and a multiple testing adjusted *p*-value below 0.05 were considered to be significantly altered. The raw sequencing data were deposited in the NCBI sequence read archive (SRA) as a study under the accession number PRJNA293284.

### Determination of the proton motive force

PA14 and PAO1 and the corresponding *hfq* deletion strains were grown in 20 ml SCFM at 37°C to an OD_600_ of 1.8–2. Then triton was added to a final concentration of 0.002% to avoid clumping. The membrane potential was measured using the dyes 3,3′-diethyloxacarbocyanine iodide [DiOC_2_(3)] (BacLight^TM^ kit; Invitrogen) and TO-PRO-3 (TO-PRO^TM^-3 Ready Flow^TM^ Reagent, Invitrogen) to distinguish dead from live cells during flow cytometry. The cultures were diluted 250-fold in buffer containing 100 mM Tris, 1 mM EDTA, and 80 mM NaCl (pH adjusted to 7.4 using HCl, Glasser et al., 2014). TO-PRO-3 was added according to the manufacturer's instructions and then DiOC_2_(3) was added in to a final concentration of 30 μM. For depolarized controls, carbonyl cyanide 3-chlorophenylhydrazone (CCCP, dissolved in DMSO, SIGMA) was added to a final concentration of 15 μM. The samples were incubated for 15 min at room temperature and then analyzed on a BD FACS-Calibur flow cytometer. DiOC_2_(3) fluorescence was measured using excitation at 488 nm and emission at 530 ± 30 nm (FL1) and 650LP nm (FL3). TO-PRO-3 fluorescence was measured using excitation at 635 nm and emission at 661 ± 13 nm (FL4). The analysis of the data was performed with FlowJo® (v10). Living cells were selected by the low fluorescence peak revealed by TO-PRO-3 staining, which were further gated on a log-scale scatter plot of side scatter (SSC) vs. forward scatter (FSC) for further analysis. Cells with a membrane potential were distinguished by an increased red/green (FL3/FL1) fluorescence ratio relative to depolarized controls. The ratiometric parameter (red-to-green ratio) was calculated according to the manufacturer's instructions [FL-3–FL-1 + 1.5^*^(#channels per decade)] and normalized to the value obtained with the wild type strain, which was set to 100%.

### C-di-GMP assay

The c-di-GMP assay was performed with strains PAO1 and PA14 and their corresponding *hfq* deletion mutants harboring plasmid pCdrA-*gfp* (ASV)^C^ (Rybtke et al., 2012). The strains were grown in 25 ml SCFM supplemented with 0.01% Triton X-100 and gentamicin (30 mg/L) for plasmid maintenance in 100 ml flasks to an OD_600_ of 1.8–2.0. The green fluorescence (arbitrary GFP units) as well as A_600_ was measured with a plate reader (Synergy H1, BioTek). In addition, PAO1, PAO1Δ*hfq*, PA14, and PA14Δ*hfq* without plasmid were grown in the same medium to determine auto-fluorescence of the strains (blank). The corresponding auto-fluorescence was then subtracted from the green fluorescence values of each biological replicate. These values were normalized to the corresponding A_600_ values.

### Biofilm assay

Static-culture biofilm assays were performed in duplicate in 5 ml round bottom polystyrene tubes (Falcon). Cells were grown in SCFM for 48 h. The biofilms were washed with water and stained with 0.1% crystal violet solution for 10 min. The dye bound, which is proportional to the biofilm produced, was solubilized with 96% (v/v) ethanol and the absorption was photometrically measured at 595 nm (A_595_).

## Results and discussion

### The susceptibility toward several classes of antibiotics is increased in *P. aeruginosa hfq* deletion strains

To gain better insights into the role of Hfq in antibiotic resistance in *Pseudomonas aeruginosa*, we first determined the minimal inhibitory concentration (MIC) of seven different classes of clinically relevant antibiotics for PAO1 and PAO1Δ*hfq* (Table 1) and for the clinical strain PA14 and the isogenic *hfq* deletion mutant PA14Δ*hfq* (Table 2). The strains were grown in SCFM, which approximates to the conditions of the CF lung. When compared to the corresponding wild-type strains, the *hfq* deletion mutants showed a higher susceptibility toward all tested antibiotics regardless of whether a high inoculum (4 × 10^9^ cells) or a low inoculum (2 × 10^6^ cells) was seeded on the plates (Tables 1, 2). The largest differences were observed with cefepime and fosfomycin, which interfere with cell wall synthesis, and with gentamicin and tetracycline, which inhibit protein synthesis. The MICs for the same antibiotics were also tested for PAO1 and PAO1Δ*hfq* in LB-medium (Supplementary Table S1). As observed in SCFM, PAO1, and PAO1Δ*hfq* showed the same pattern of sensitivity toward the studied antibiotics. However, as these growth-dependent assays could reflect the impact of the *hfq* deletion on the ability of the respective strains to grow and divide, we also tested whether an increased susceptibility to an antibiotic is observed with PA14Δ*hfq* grown to early stationary phase. PA14 and PA14Δ*hfq* were grown in SCFM to an OD_600_ of 2.0. Then, to aliquots of 180 μl culture either 20 μl of SCFM or 20 μl of SCFM containing gentamicin to a final concentration of 2 mg/L were added. For CFU determination, samples were withdrawn at an OD_600_ of 2.0 (initial inoculum) and after 18 h of incubation in the presence or absence of 2 mg/L gentamicin. When compared to the wild type, the *hfq* deletion mutant showed a 10–fold reduction in the CFU (Supplementary Figure S1). Thus, the increased sensitivity of the *hfq* deletion mutants appeared to be independent of growth.

**Table 1:** Susceptibility of PAO1 and PAO1Δ*hfq* toward different classes of antibiotics in SCFM medium.

**Classes/Subclass**	**Antibiotic**	**Mode of action**	**MIC**	**PAO1**	**PAO1Δ*hfq***	**PAO1**	**PAO1Δ*hfq***
**SCFM**				**High inoculum**^a^		**Low inoculum**^b^	
β**-LACTAM ANTIBIOTICS**							
Cephems/Cephalosporins IV	Cefepime	Cell wall	mg/L	2/3	0.75/0.75	1/1	0.5/0.5
Penems/Carbapenems	Imipenem	Cell wall	mg/L	1/1	0.5/0.5	1/1	0.5/0.5
**NON-**β**-LACTAM ANTIBIOTICS**							
Aminoglycosides	Gentamicin	Protein synthesis	mg/L	8/8	2/2	16/8	2/2
Fluoroquinolones	Ciprofloxacin	DNA metabolism	mg/L	0.25/0.25	0.12/0.12	0.12/0.12	0.06/0.12
Fosfomycins	Fosfomycin	Cell wall	mg/L	>1024/>1024	64/64	>1024/>1024	4/2
Lipopeptides/Polymixins	Colistin	Cell membrane	mg/L	3/4	2/3	6/4	3/2
Tetracyclines	Tetracycline	Protein synthesis	mg/L	128/128	32/32	16/16	8/8

*/, Results of two independent experiments; >, more resistant than the highest concentration tested*.

a *High inoculum, corresponds to 4 × 10^9^ CFU/mL*.

b *Low inoculum, corresponds to 2 × 10^6^ CFU/mL*.

**Table 2:** Susceptibility of PA14 and PA14Δ*hfq* toward different classes of antibiotics in SCFM medium.

**Classes/Subclass**	**Antibiotic**	**Mode of action**	**MIC**	**PA14**	**PA14Δ*hfq***	**PA14**	**PA14Δ*hfq***
**SCFM**				**High inoculum**^a^		**Low inoculum**^b^	
β**-LACTAM ANTIBIOTICS**							
Cephems/Cephalosporins IV	Cefepime	Cell wall	mg/L	2/3	1.5/1.5	0.75/0.75	0.5/0.5
Penems/Carbapenems	Imipenem	Cell wall	mg/L	1/1	0.5/0.5	0.5/1	0.25/0.25
**NON-**β**-LACTAM ANTIBIOTICS**							
Aminoglycosides	Gentamicin	Protein synthesis	mg/L	8/8	2/2	4/4	2/1
Fluoroquinolones	Ciprofloxacin	DNA metabolism	mg/L	0.25/0.25	0.12/0.12	0.12/0.06	0.06/0.06
Fosfomycins	Fosfomycin	Cell wall	mg/L	>1024/>1024	12/6	384/384	1/2
Lipopeptides/Polymixins	Colistin	Cell membrane	mg/L	12/12	4/4	2/3	1.5/1.5
Tetracyclines	Tetracycline	Protein synthesis	mg/L	32/32	8/16	64/64	16/16

*/, Results of two independent experiments; >, more resistant than the highest concentration tested*.

a *High inoculum, corresponds to 4 × 10^9^ CFU/mL*.

b *Low inoculum, corresponds to 2 × 10^6^ CFU/mL*.

### Hfq controls functions associated with antibiotic resistance

**Figure 1:**
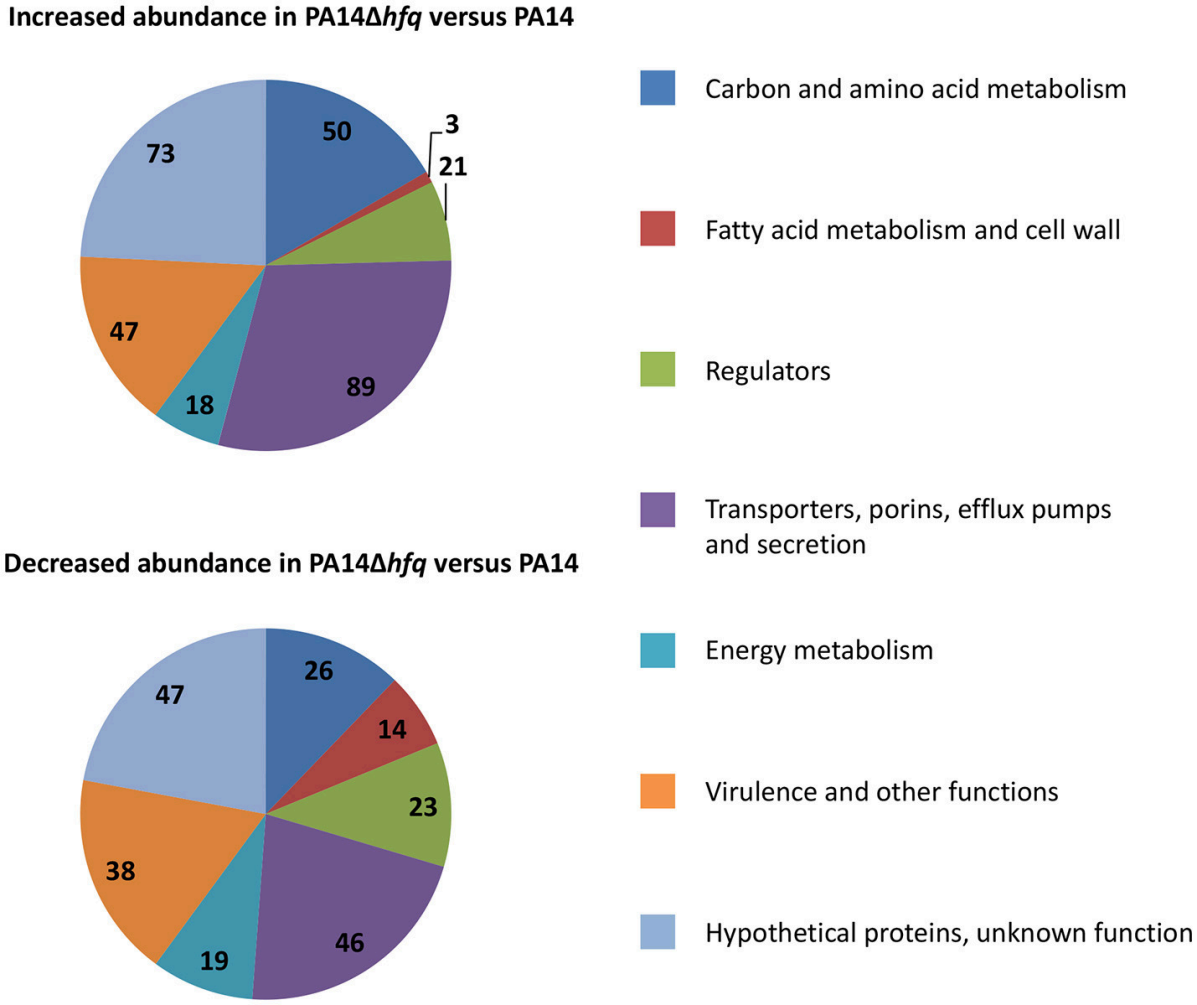
Functional classification of transcripts with increased **(upper panel)** or decreased **(lower panel)** abundance in PA14Δ*hfq* when compared with PA14. The numbers represent the total amount of transcripts in each category with the color code shown at the right.

Next, we asked whether Hfq impacts on transcripts the regulation or mutation of which can interfere with the susceptibility to different antibiotics. As the sensitivity pattern of the wild-type and *hfq* mutant strains toward the different antibiotics was independent of the inoculum size (Tables 1, 2), a comparative RNA_Seq_ based transcriptome analysis was performed with PA14 and PA14Δ*hfq* grown in SCFM to an OD_600_ of 1.8–2.0 (early stationary phase). A *P*-value (adjusted for multiple testing) of 0.05 was set as a threshold for significance. To select predominantly for transcripts that are regulated by Hfq, only transcripts with a change in abundance (fold-change) of ± 4 were considered. Applying these criteria, 301 transcripts were up-regulated and 213 transcripts were down-regulated in PA14Δ*hfq* when compared with PA14 (Supplementary Tables S2, S3). Next, the transcripts were clustered into 7 different categories (Figure 1) based on previous functional characterizations in *P. aeruginosa* or similar characterized genes in other bacteria using the Pseudomonas genome database (Winsor et al., 2016). Genes encoding hypothetical proteins within operons (Wurtzel et al., 2012) with partly known functions were placed together. One of the largest group (23% in total) represented transcripts encoding “hypothetical proteins with unknown function.”

50 transcripts with increased and 28 transcripts with decreased abundance are related to “carbon and amino acid metabolism.” The high number of up-regulated genes within this category can be rationalized with Hfq being the major regulator of CCR in *P. aeruginosa* (Sonnleitner and Bläsi, 2014). It has been reported that C-sources can affect antibiotic resistance (Walker and Durham, 1975; Conrad et al., 1979; Barraud et al., 2013). However, the molecular mechanism underlying this phenomenon remained largely elusive. Meylan et al. (2017) suggested a link between central carbon metabolism and aminoglycoside sensitivity. While fumarate potentiated tobramycin susceptibility, glyoxylate promoted tolerance. This was explained by either activating (fumarate) or repressing (glyoxylate) the TCA cycle, resulting in differences in the proton motive force (PMF), and thus differences in tobramycin uptake. Our RNA_Seq_ analysis revealed elevated transcript levels of *sdhC/D*, encoding succinate dehydrogenase, *acnB*, encoding aconitate hydratase, and *acsL*, encoding acetyl-CoA synthetase, in PA14Δ*hfq* when compared with PA14, which might suggest an up-regulation of the TCA cycle in PA14Δ*hfq* (Supplementary Table S2).

Related to carbon metabolism, 3 and 14 up- and downregulated genes, respectively, clustered in the category “fatty acid metabolism and cell wall.” Among those *rfaD*, an ortholog of *Burkholderia cenocepacia hldD* gene, was 237.9-fold down-regulated in PA14Δ*hfq* when compared with PA14 (Supplementary Table S3). The *rfaD*/*hldD* functions are involved in modification of heptose sugars prior to their incorporation into the LPS core (Raetz and Whitfield, 2002). It was shown for *B. cenocepacia* that deletion of *hldD* together with *hldA* resulted in short lipidA-core oligosaccharides and higher sensitivity to polymyxin B (Loutet et al., 2006). In addition, *orfM* and *orfN*, which are orthologs of PAO1 *wbpK* and *wbpL*, showed a 6.7 and 6.2-fold reduced abundance, respectively, in the absence of Hfq (Supplementary Table S3). They encode proteins important for the initial step of O-polysaccaride biosynthesis. In *E. coli* the lack of the O-antigen chain of LPS results in higher sensitivity toward hydrophobic antibiotics, like erythromycin and rifampicin, as well as to hydrophilic antibiotics, like vancomycin (Hirvas et al., 1997). Furthermore, a *wbpL* mutant of *P. aeruginosa* showed increased outer membrane permeability for the antimicrobial peptide indolicidin (Gooderham et al., 2008). One positive regulator of *wbpL* transcription is PsrA (Gooderham et al., 2008). The transcript abundance of *psrA* was 4.2-fold reduced in PA14Δ*hfq* when compared with PA14 (Supplementary Table S3), which is in line with the deregulation of the *wbpL* gene. A *psrA* mutant exhibit increased susceptibility to polymyxin B (Gooderham et al., 2008), which is in agreement with a higher sensitivity to colistin in PA14Δ*hfq* when compared with PA14.

The category with the highest number of transcripts belongs to “transporters, porins, efflux pumps, and secretion” comprising 135 transcripts, 89 and 46 of which were up- and down-regulated, respectively. Intrinsic resistance to antibiotics is mainly brought about by efflux pumps (Li et al., 1994). However, the corresponding genes encoding efflux pumps such as MexAB-OprM, MexCD-OprJ, MexEF-OprN or MexXY-OprM, which are known to be involved in resistance toward several antibiotics (Morita et al., 2001; Hocquet et al., 2006; Llanes et al., 2011; Peng et al., 2017), did not show in the RNA_Seq_ analysis.

The susceptibility toward imipenem increased ~8 and ~16-fold in PA14Δ*hfq* and PAO1Δ*hfq* when compared to the respective parental strains (Tables 1, 2). The transcript abundance of *oprD*, encoding a porin responsible for carbapenem uptake (Lynch et al., 1987) was 5-fold higher in PA14Δ*hfq* than in PA14 (Supplementary Table S2). It has been previously reported that Hfq negatively controls *oprD* expression indirectly on the transcriptional and directly on the post-transcriptional level via sRNAs (Ducret et al., 2016; Zhang et al., 2017), which can explain the increased susceptibility of the *hfq* deletion strains toward imipenem (Tables 1, 2, Supplementary Table S1). Moreover, the Hfq-Crc complex appears to repress translation of *oprD* mRNA directly (P. Pusic, unpublished). This seems to be also reflected in the observation that the OprD levels were elevated in a PAO1 *crc* mutant, which was paralleled by an increased susceptibility to meropenem and imipenem (Linares et al., 2010).

Another striking result was obtained with fosfomycin. PA14 was resistant to fosfomycin (Table 2), whereas the PA14Δ*hfq* strain was sensitive. The glycerol-3-phosphate permease GlpT was shown to be the only fosfomycin transporter in *P. aeruginosa* (Castañeda-García et al., 2009). The *glpT* transcript was 4.4-fold upregulated in PA14Δ*hfq* when compared with PA14 (Supplementary Table S2), suggesting an elevated uptake of fosfomycin in PA14Δ*hfq* and increased susceptibility toward fosfomycin (Table 2). The negative regulation of *glpT* by Hfq was evident from the result obtained with a transcriptional *glpT*-*lacZ* reporter gene. The β-galactosidase activity conferred by the transcriptional *glpT-lacZ* fusion was increased approximately 1.5-fold in PA14Δ*hfq* when compared with PA14 (Supplementary Figure S2), suggesting an indirect effect of Hfq. A PAO1*crc-* mutant showed as well an increased sensitivity to fosfomycin and increased levels of the *glpT* transcript (Linares et al., 2010), indicating that Hfq together with Crc might control a factor involved in *glpT* expression.

Several genes important for iron uptake including the transcriptional regulator gene *pvdS* are differentially abundant in PA14Δ*hfq* when compared with PA14 (Supplementary Tables S2, S3). For example *fpvA* and *fpvB*, encoding ferropyoverdine receptors, as well as the siderophore receptor PA14_45540 were upregulated in PA14Δ*hfq*, whereas pyochelin biosynthesis and uptake genes as well as *feoAB*, encoding ferrous iron transport proteins, were down-regulated when compared with PA14, suggesting aberrant iron uptake in PA14Δ*hfq*. It has been previously shown that iron depletion in *P. aeruginosa* increases the susceptibility to certain antibiotics (Oglesby-Sherrouse et al., 2014). However, it was surprising to find many iron uptake genes to be regulated by Hfq in SCFM, as the iron concentration was 100 μM. The differential abundance of functions involved in iron metabolism in PA14Δ*hfq* might be ascribed to differential c-di-GMP levels (Frangipani et al., 2014). On the other hand, there is a link between iron availability, oxidative stress and redox state of the cell (Cornelis et al., 2011), which might be also affected by Hfq, especially since transcripts like *sodB* and *katE*, encoding superoxide dismutase and catalase, were more abundant in PA14Δ*hfq* than in PA14 (Supplementary Table S2).

The category “energy metabolism” comprises 18 and 19 transcripts with increased and decreased abundance, respectively, in PA14Δ*hfq* when compared with PA14. In the absence of Hfq, the *coxBAG-coIII* operon (encoding aa_3_-type cytochrome c oxidase) is up-regulated and the *cyoABCDE* operon (encoding bo_3_-type quinol oxidase) and *ccoN2O2P2* (encoding cbb_3_-type cytochrome oxidase 2) are down-regulated (Supplementary Tables S2, S3). The expression of the *coxBAG-coIII* operon is low under normal growth conditions and is induced during carbon, nitrogen and iron starvation (Kawakami et al., 2010). The *cox* promoter was found to be activated by the stationary phase sigma factor RpoS and repressed by the two component system RoxS/R (Schuster et al., 2004; Kawakami et al., 2010). As the RNA_Seq_ analysis revealed increased transcript levels of *rpoS* in PA14Δ*hfq* when compared with PA14 (Supplementary Table S2), it could explain the increased expression of the *cox* operon. On the other hand, Hfq might also repress translation of the *cox* operon directly. A potential Hfq binding site (-//5′-AAAAACAACGATAAG-3′//-) is present upstream of the *coxB* start codon. The aa_3_-type cytochrome c oxidase has the highest proton pumping activity, and if up-regulated under nutrient rich conditions, *cox* over-expression might cause an imbalance of energy and redox homeostasis (Arai, 2011). Aminoglycoside uptake is considered to be an energy-dependent process that requires both a high membrane potential and electron flow through the respiratory chain (Taber et al., 1987). Disruption of respiration or uncoupling of the electron transport chain (ETC) protects bacteria against aminoglycoside treatment (Bryan and Kwan, 1983; Taber et al., 1987; McCollister et al., 2011). Enhanced TCA cycle activity as well as upregulation of the *cox*-operon in the PA14Δ*hfq* strain might thus suggest an increased respiration and PMF and thus elevated aminoglycoside uptake and consequently gentamicin susceptibility.

The last category of transcripts affected by Hfq includes genes related to “virulence and other functions,” some of which might be important for pathogenicity of *P. aeruginosa*, e.g., *rhlAB*, encoding rhamnosyltransferase (Abdel-Mawgoud et al., 2010) and several phenanzine biosynthesis genes (Pierson and Pierson, 2010; Mavrodi et al., 2013, Supplementary Tables S2, S3).

### Reduced energy metabolism in the absence of Hfq

**Figure 2:**
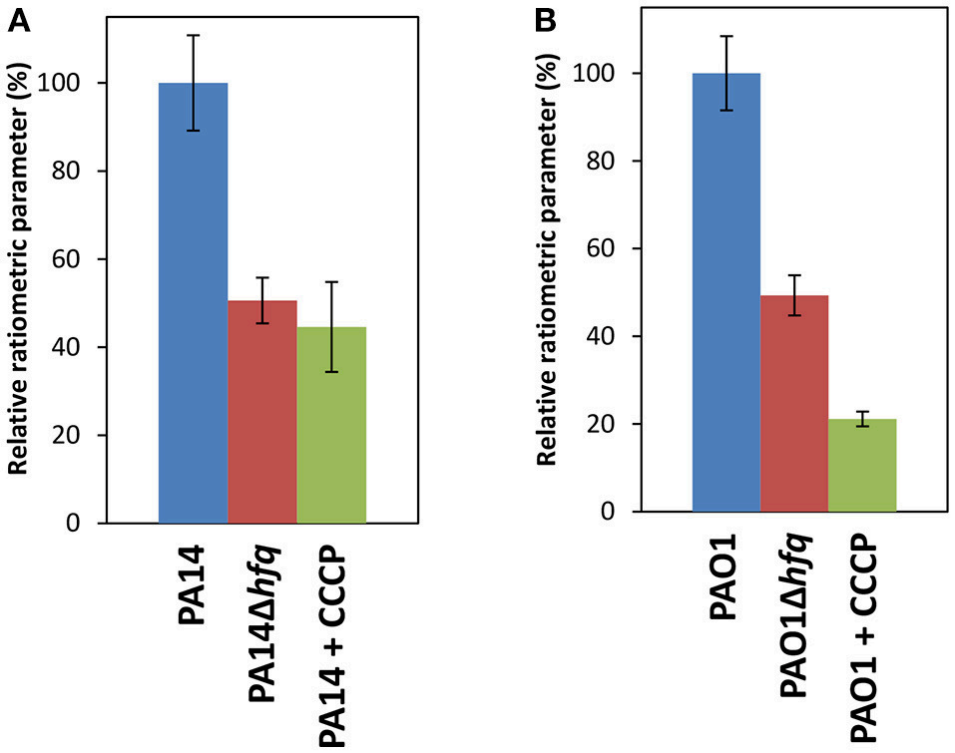
Reduced membrane potential in the absence of Hfq. Determination of the proton motive force in PA14 **(A)** and in PAO1 **(B)** and their corresponding *hfq* deletion strains using the BacLight^TM^ kit. The strains were grown in SCFM to an OD_600_ of 1.8–2.0. The analysis was performed as described in Materials and Methods. The wild type strains and the corresponding *hfq* deletion strains are indicated by blue and red bars, respectively. Membrane depolarization in PA14 (**A**; green bar) and in PAO1 (**B**, green bar) was elicited with the addition of CCCP. The experiments were performed in duplicates. Error bars are given as standard deviation of the means over the populations of the biological replicates.

The RNA_Seq_ analysis suggested an increased PMF in PA14Δ*hfq* due to the up-regulation of the *cox* operon (Supplementary Table S2). The aa_3_-type oxidase translocates the highest number of protons across the cell membrane per oxygen atom consumed (6 H^+^/O ratio) (Arai, 2011), which might allow for a partial compensation of the down-regulated operons encoding bo_3_ and ccb_3_2-type oxidases (4 H^+^/O ratio) (Supplementary Table S3). To test whether Hfq affects the PMF and therefore the membrane potential, we used a flow cytometry based method for membrane potential measurements. This technique is based on the carbocyanine dye DiOC_2_(3) (3,3′-diethyloxacarbocyanine iodide). DiOC_2_(3) exhibits green fluorescence in all bacterial cells, but the fluorescence shifts toward red emission as soon as the dye molecules self-associate due to higher cytosolic concentrations that is caused by an increased membrane potential. For a better correlation between dye uptake and membrane potential, ratiometric values (the determined values of the fluorescence were divided by a parameter value representative for cell sizes) were established (Novo et al., 1999). Unexpectedly, PA14Δ*hfq* revealed a 50% reduced ratiometric value compared to PA14, which was similar to that when the membrane potential was disrupted by carbonyl cyanide 3-chlorophenylhydrazone (CCCP) (Figure 2A). Comparable results were obtained with the corresponding PAO1 strains (Figure 2B), except that the impact of CCCP was more pronounced (80% reduction). These results indicated that deletion of *hfq* results in a decrease of the energy metabolism, which however cannot be straightforwardly reconciled with the RNA_Seq_ analysis. However, RNA_Seq_ based transcriptome studies only reveal either direct or indirect transcriptional regulation by Hfq or reflect post-transcriptional regulation if the regulatory event impacts on transcript abundance. Thus, as shown by Grenga et al. (2017), a considerable proportion of the Hfq regulon that is controlled at the translational and post-translational level will not be reflected in the transcriptome analysis.

It has been shown that disruption of the membrane potential by CCCP resulted in increased susceptibility to colistin and ciprofloxacin in some clinical strains of *P. aeruginosa* (Adabi et al., 2015; Ni et al., 2016) and that the PMF is important for the function of MFS (major facilitator superfamily) and RND (resistance-nodulation-division) efflux pumps (Paulsen et al., 1996). Thus, the reduced PMF in the *hfq* deletion strains might interfere with antibiotic efflux, resulting in increased susceptibility to certain antibiotics, like β-lactams, fluoroquinolones, tetracycline and aminoglycosides that are exported via efflux pumps (Schweizer, 2003). The apparent reduction in energy metabolism of the *hfq* deletion strains (Figure 2) cannot be easily reconciled with the increased susceptibility to gentamicin in terms of drug uptake. However, in addition to a possible impairment of the efflux pumps, the down-regulation of some LPS biosynthetic genes in PA14Δ*hfq* (Supplementary Table S3) could as well contribute to the increased susceptibility toward aminoglycosides (Hirvas et al., 1997).

### Reduced c-di-GMP levels in the absence of Hfq

**Figure 3:**
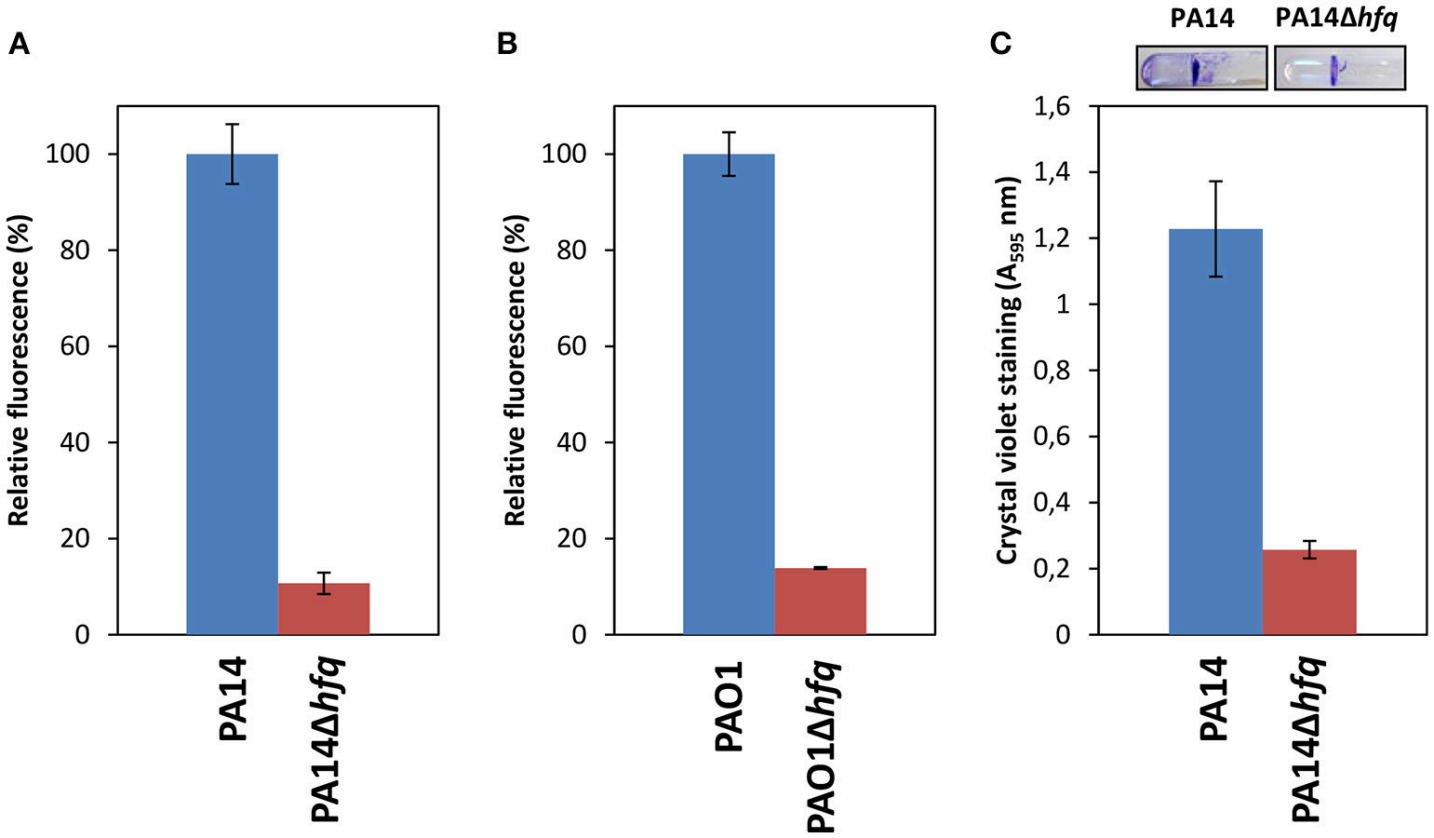
Deletion of *hfq* results in reduced c-di-GMP levels and biofilm formation. PA14 **(A)** and PAO1 **(B)** (blue bars) and the corresponding *hfq* deletion strains (red bars) harboring plasmid pCdrA::*gfp*(ASV)^c^, carrying a c-di-GMP sensitive transcriptional *cdrA*-*gfp* fusion gene, were grown in SCFM to an OD_600_ of 1.8–2.0. The analysis was performed as described in Materials and Methods. **(C)** Static biofilms of PA14 and PA14Δ*hfq* were stained with crystal violet after growth in SCFM for 48 h. The stain was then eluted by ethanol and the absorption at 595 nm was measured. Representative pictures of the PA14 and PA14Δ*hfq* biofilms are shown on top. The experiments were performed in duplicate. Error bars are given as standard deviation.

Bacterial biofilms are more resistant to antibiotics than planktonically growing cells (Ceri et al., 1999; Olsen, 2015). Most likely, there is a multitude of factors that promote biofilm-specific antibiotic tolerance/resistance (Olsen, 2015). The secondary messenger c-di-GMP is a key signal in post-transcriptional regulation of biofilm formation and coordinates the transition from a motile to a sessile lifestyle and *vice versa* (i.e., dispersion, Valentini and Filloux, 2016; Moradali et al., 2017). A comparison of the RNA_Seq_ analysis performed in this study with the transcriptome profiles of *P. aeruginosa* subjected to high or low c-di-GMP levels (Lin Chua et al., 2017) revealed that 43% of all Hfq regulated transcripts are also c-di-GMP dependent. To determine whether Hfq impacts on the c-di-GMP levels, a transcriptional reporter gene was employed that is comprised of the c-di-GMP responsive *cdrA* promoter fused to the *gfp* (ASV)^C^ gene, which encodes an unstable GFP variant (Rybtke et al., 2012). As shown in Figure 3A, under these conditions, the reporter gene conferred ~ 60% less GFP activity in PA14Δ*hfq*(pCdrA-*gfp*(ASV)^C^) when compared with PA14 (pCdrA-*gfp*(ASV)^C^). A similar reduction in GFP activity of 70% was obtained with PAO1Δ*hfq* (pCdrA-*gfp*(ASV)^C^) when compared with PAO1(pCdrA-*gfp*(ASV)^C^) (Figure 3B). These results showed that the c-di-GMP levels were reduced in the *hfq* deletion strains, which was also reflected by the diminished ability of PA14Δ*hfq* to form biofilms in SCFM when compared to PA14 (Figure 3C).

Reduced c-di-GMP levels resulted in a mild increase of the OprD protein levels (Nicastro et al., 2014). Thus, in addition to the Hfq-mediated negative riboregulation of *oprD* (Zhang et al., 2017), the decreased c-di-GMP levels in the *hfq* deletion strains could as well contribute to the increased imipenem susceptibility. The transcriptome analysis did not identify a differential abundance in the absence of Hfq of transcripts encoding a c-di-GMP phosphodiesterase or a diguanylate cyclase. However, as discussed above, the RNA_Seq_ analysis has limitations (Grenga et al., 2017) in that translational and/or post-translational regulatory events might not be mirrored.

### Harnessing metabolic control for sensitization toward antibiotics

As the deletion of *hfq* resulted in increased susceptibility of *P. aeruginosa* to different antibiotics, we next asked whether we could harness the observed interference by CrcZ RNA of Hfq-mediated regulatory events (Pusic et al., 2016; Sonnleitner et al., 2017), i.e., whether an up-regulation of CrcZ RNA results in increased antibiotic susceptibility through titration of Hfq.

**Figure 4:**
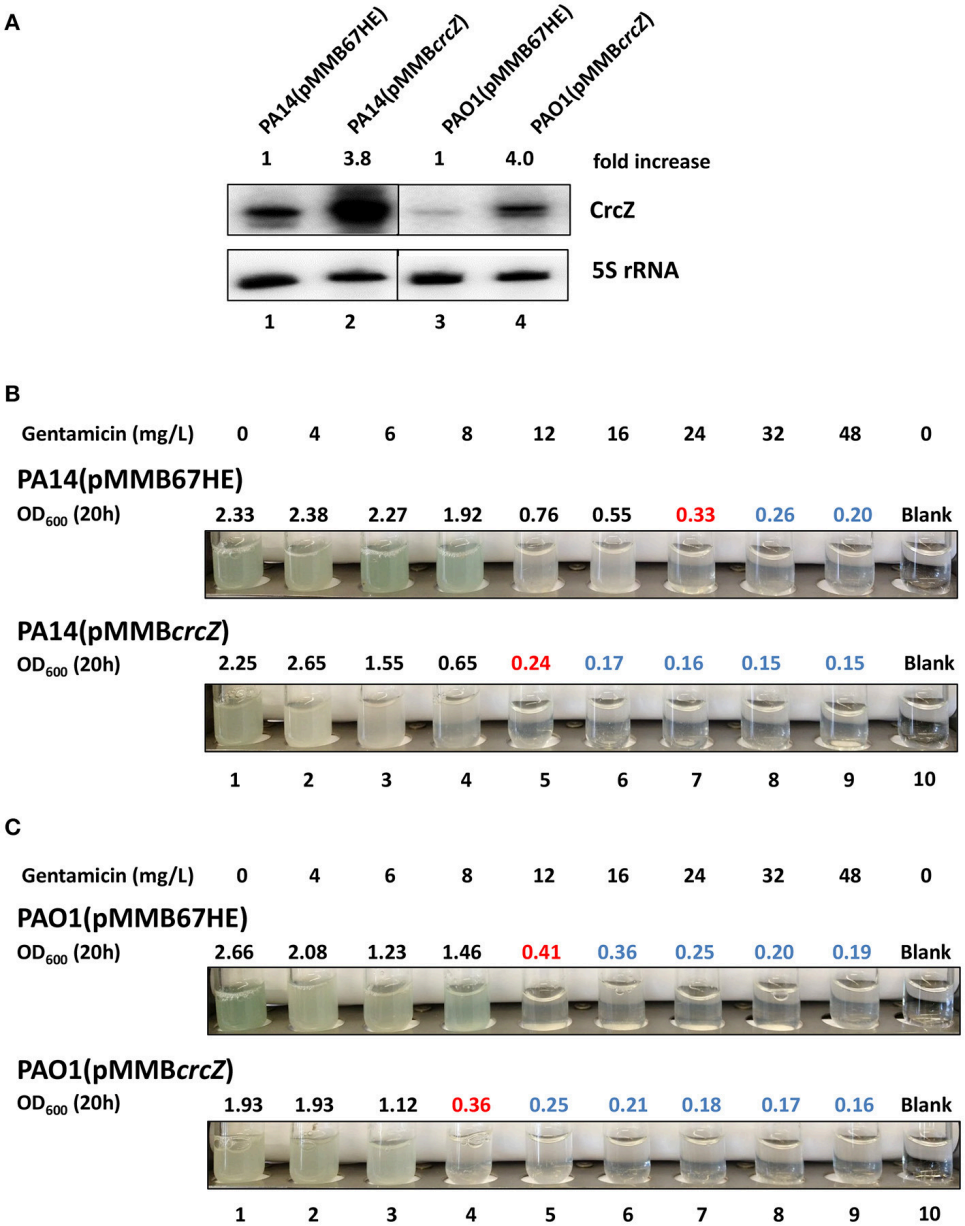
Over-production of CrcZ results in increased susceptibility to gentamicin. **(A)** PA14 and PAO1 harboring plasmid pMMB67HE and plasmid pMMB*crcZ*, respectively, were grown in SCFM for 20 h. The cells were harvested and the CrcZ levels were determined by Northern-blot analysis. 5S rRNA served as a loading control. The fold increase was calculated by dividing the signal intensity of CrcZ by the corresponding signal of 5S rRNA and normalized to the signal obtained with the respective wild type strain harboring plasmid pMMB67HE that was set to 100%. Lanes 1 and 2 contain RNA extracted from strains PA14 (pMMB67HE) and PA14 (pMMB*crcZ*), respectively. Lanes 3 and 4 contain RNA extracted from strains PAO1(pMMB67HE) and PAO1(pMMB*crcZ*), respectively. **(B,C)** MIC determination for gentamicin of PA14 **(B)** and PAO1 strains **(C)**. The cells harboring plasmid pMMB67HE (upper panels) and plasmid pMMB*crcZ* (lower panels), respectively, were grown as described above. The different concentrations of gentamicin added are indicated on top. Pictures were taken and the OD_600_ was measured 20 h after inoculation. The antibiotic concentrations in the presence of which the cells did not grow above OD_600_ of 0.5 (marked in red) were considered as MIC. All OD_600_ values above this gentamicin concentration are depicted in blue indicating toxicity. The experiments were performed in duplicate, revealing the same MICs. Only one representative experiment is shown.

To address this, PA14 and PAO1 harboring plasmid pMMB67HE (empty plasmid) and plasmid pMMB*crcZ* (P_*tac*_ driven constitutive expression of *crcZ*), respectively, were grown in SCFM medium to an OD_600_ of 1.0. Then, one ml of culture was mixed with one ml of fresh SCFM containing serial dilutions of gentamicin and incubated for 20 h at 37°C. As shown in Figure 4, over-production of CrcZ (Figure 4A) resulted in an increased sensitivity of PA14 (pMMB*crcZ*) (Figure 4B) and PAO1(pMMB*crcZ*) (Figure 4C) toward the antibiotic in a manner comparable with the patterns observed for the *hfq* deletion strains (Tables 1, 2).

The *crcZ* promoter activity, and thus the levels of CrcZ are increased when the cells grow on non-preferred carbon sources such as oxaloacetate (OAA) or histidine (Valentini et al., 2014). To further test whether the CrcZ levels can be correlated with antibiotic susceptibility, the growth inhibition zones obtained with PA14 and PAO1 in the presence of cefepime, colistin, ciprofloxacin and gentamicin were determined on BSM agar plates supplemented with either succinate or OAA. When compared to cells growing on succinate, PAO1 and PA14 growing on OAA were more susceptible to all tested antibiotics (Table 3), supporting the hypothesis that C-source dependent antibiotic susceptibility can be linked to increased CrcZ levels and thus enhanced sequestration of Hfq.

**Table 3:** The sensitivity toward several antibiotics is increased during growth on oxaloacetate.

**Antibiotic**	**Conc.(μg)**	**PA14 (%)**		**PAO1 (%)**	
**BSM**		**Succinate**	**Oxaloacetate**	**Succinate**	**Oxaloacetate**
Cefepime	60	100 ± 6	107 ± 6	100 ± 3	137 ± 3
Colistin	25	100 ± 5	130 ± 6	100 ± 5	126 ± 6
Ciprofloxacin	5	100 ± 5	112 ± 4	100 ± 3	125 ± 2
Gentamicin	120	100 ± 0	119 ± 0	100 ± 3	131 ± 4

*The diameter of the growth inhibition zone for each antibiotic in BSM-succinate was set to 100 %. The inhibition zones obtained during growth on oxaloacetate were normalized to the diameter revealed in BSM-succinate. The data are derived from two independent experiments as mean ± standard deviation*.

**Figure 5:**
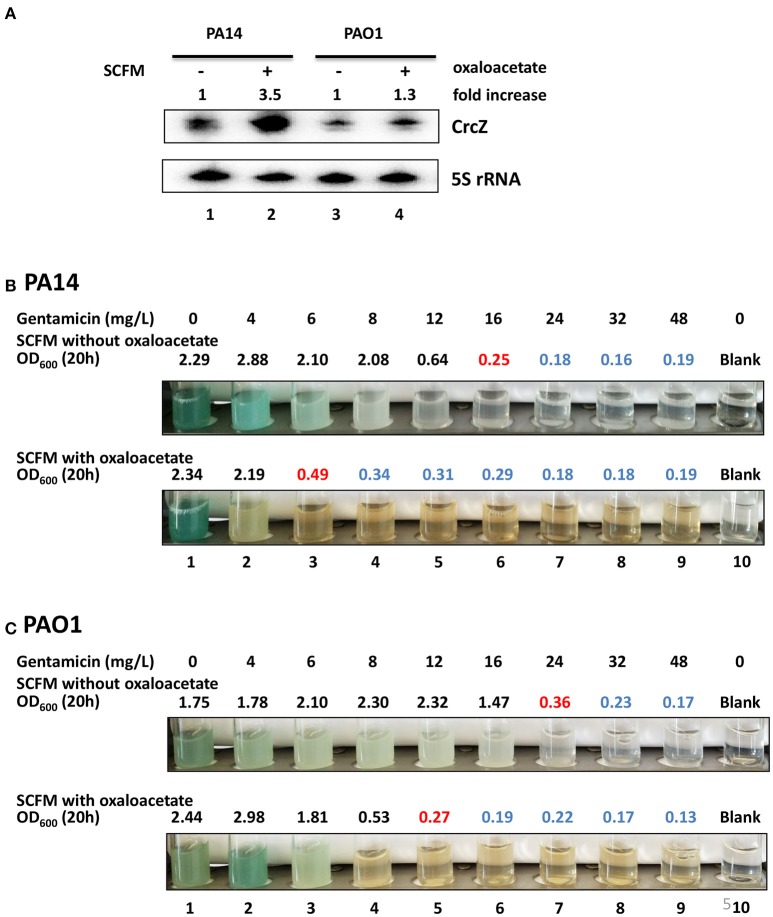
Addition of OAA to SCFM results in increased CrcZ levels and in increased sensitivity toward gentamicin. **(A)** PA14 and PAO1 cultures were diluted to an initial OD_600_ of 0.5 and incubated for 20 h in SCFM with or without 40 mM OAA. The CrcZ levels were determined by Northern-blot analysis. 5S rRNA served as a loading control. The fold increase was calculated by dividing the signal intensity of CrcZ by the corresponding signal of the 5S rRNA and normalized to the signal without OAA that was set to 100%. Lanes 1 and 2, contain RNA extracted from PA14 in the presence and absence of OAA, respectively; lanes 3 and 4, contain RNA extracted PAO1 in the presence and absence OAA, respectively. **(B,C)** MIC determination for gentamicin of PA14 **(B)** and PAO1 **(C)**. The cells were grown as described above in the absence (upper panel) and in the presence (lower panel) of OAA. The different concentrations of gentamicin added are indicated on top. Pictures were taken and the OD_600_ was measured 20 h after inoculation. The antibiotic concentrations in the presence of which the cells did not grow above OD_600_ of 0.5 (marked in red) were considered as MIC. All OD_600_ values above this gentamicin concentration are depicted in blue indicating toxicity. The experiments were performed in duplicate, revealing the same MICs. Only one representative experiment is shown.

Next, we asked whether the CrcZ levels and thus antibiotic susceptibility can be increased in complex medium by adding OAA. PA14 and PAO1 were grown in SCFM medium to an OD_600_ of 1.0. Then, one ml of culture was mixed with one ml of fresh SCFM with or without 40 mM OAA and incubated for 20 h at 37°C. As shown in Figure 5A, the levels of CrcZ increased upon addition of OAA to SCFM in PA14 and PAO1. Concurrently, the MIC for gentamicin changed from 16 mg/L (without OAA) to 6 mg/L (with OAA) in PA14 (Figure 5B) and from 24 mg/L (without OAA) to 12 mg/L (with OAA) in PAO1 (Figure 5C), indicating a significant increase in susceptibility for gentamicin upon addition of OAA. A similar increase in the susceptibility toward cefepime was also observed for PAO1 when OAA was added to the growing culture (change in the MIC from 2,500 to 310 mg/L in the presence of OAA, Supplementary Figure S3). Taken together, these data hold promise that induced synthesis of CrcZ can be harnessed to increase the susceptibility of or re-sensitize *P. aeruginosa* toward different antibiotics by means of sequestration of Hfq.

## Conclusions and perspectives

In this study we have shown that Hfq impacts on energy metabolism and c-di-GMP levels. Thus, the absence of Hfq seems to impose a general fitness burden which is also apparent from the somewhat impaired growth of the *hfq* deletion strains (not shown). It was shown that dormant *P. aeruginosa* exhibit an impaired aminoglycoside uptake. This phenomenon called phenotypic tolerance is related to persistence of bacteria after antibiotic treatment (Martínez and Rojo, 2011). Interestingly, *P. aeruginosa* can be re-sensitized toward aminoglycosides by metabolic stimulation (Meylan et al., 2017). However, in contrast to persister cells, the slower growing *hfq* deletion strains were more susceptible to all tested antibiotics including aminoglycosides.

The transcriptome analysis revealed that Hfq governs the expression of many functions, which affect antibiotic susceptibility. Thus, physiological changes on the one hand and the regulation of specific antibiotic resistance determinants on the other hand seem to collectively contribute to the observed increased susceptibility to antibiotics. The specific regulatory mechanisms exerted by Hfq on resistance genes/determinants being it via riboregulation or direct translational repression remain to be elucidated for the individual functions in detail. However, it is striking that a PAO1 *crc*-deficient mutant mirrored the increased susceptibility phenotypes of the PAO1Δ*hfq* strain in terms of increased sensitivity toward aminoglycosides, beta-lactams, fosfomycin and polymyxin B (Linares et al., 2010). Hence, it is conceivable that Hfq in conjunction with Crc directly regulates several functions conferring susceptibility at the post-transcriptional level. Thus, the Hfq-Crc complex appears to be a promising drug target to achieve a higher efficacy of treatments with a broad spectrum of different antibiotics.

Hfq is titrated by the regulatory RNA CrcZ, which abrogates its function in Hfq-Crc-mediated translational repression during CCR as well as in riboregulation (Sonnleitner and Bläsi, 2014; Sonnleitner et al., 2017). Here, we have provided a proof of principle that metabolic rewiring by induced synthesis of CrcZ RNA during growth in complex medium can increase the efficacy of gentamicin and cefepime. It is currently unknown, which signal is sensed by the CbrA/B two component system that controls CrcZ synthesis. The elucidation of the signal might thus permit induced synthesis of CrcZ in the presence of a given antibiotic. This might not only allow to lower the effective dose of antibiotics but also to re-sensitize *P. aeruginosa* strains toward certain antibiotics, e.g., for fosfomycin. In this context it is worth noting that PAO1 was recently shown to be more susceptible to fosfomycin under hypoxic conditions due to overexpression of *glpT* in an ANR dependent manner (Hirakawa et al., 2018). As CrcZ was the predominant RNA bound to Hfq in anoxic biofilms (Pusic et al., 2016) and Hfq impacts on *glpT* expression (Supplementary Figure S2) both might as well contribute to anaerobic regulation of *glpT*. Moreover, we have shown that the absence of Hfq results in less efficient aerobic (this study) and anoxic biofilm formation (Pusic et al., 2016). Thus, it appears worthwhile testing whether inactivation of Hfq might additionally increase the susceptibility of biofilms toward antibiotics.

## Author contributions

UB, ES: Conceived and designed the experiments; ES, PP, BK, DH, and AR: Performed the experiments; ES, PP, MW, and UB: Analyzed the data; ES, PP, and UB: Wrote the paper.

### Conflict of interest statement

The authors declare that the research was conducted in the absence of any commercial or financial relationships that could be construed as a potential conflict of interest.
