# Supplementary material for: Harnessing Metabolic Regulation to Increase Hfq-Dependent Antibiotic Susceptibility in Pseudomonas aeruginosa
Source: Front Microbiol. 2018 Nov 9;9:2709. doi: 10.3389/fmicb.2018.02709 (PMC6237836; doi:10.3389/fmicb.2018.02709)
Supplement: Supplementary file 3 [file Data_Sheet_1.pdf]

Supplementary Material

Harnessing Metabolic Regulation to increase Hfq-dependent Antibiotic Susceptibility in *Pseudomonas aeruginosa*

Petra Pusic[+], Elisabeth Sonnleitner*[+], Beatrice Krennmayr, Dorothea A. Heitzinger, Michael T. Wolfinger, Armin Resch and Udo Bläsi*

[+] These authors contributed equally to this work

*** Correspondence:**

Elisabeth Sonnleitner: elisabeth.sonnleitner@univie.ac.at

Udo Bläsi: udo.blaesi@univie.ac

1 Supplementary Figures and Tables

1.1 Supplementary Tables

Supplementary Table S1. Susceptibility of PAO1 and PAO1Δ*hfq* towards different classes of antibiotics in LB medium.

**Classes/Subclass**	**Antibiotic**	**Mode of action**	**MIC**	**PAO1**	**PAO1Δ*hfq***	**PAO1**	**PAO1Δ*hfq***
**SCFM**				**High inoculum[a]**		**Low inoculum[b]**	
**β-Lactam antibiotics**							
Cephems/Cephalosporins IV	Cefepime	cell wall	mg/L	1.5/1.5	1.5/1.5	0.5/0.38	0.5/0.38
Penems/Carbapenems	Imipenem	cell wall	mg/L	2/2	1/1	1/1	0.5/0.25
**Non-β-Lactam antibiotics**							
Aminoglycosides	Gentamicin	protein synthesis	mg/L	4/4	0.25/0.5	2/2	0.5/0.25
Fluoroquinolones	Ciprofloxacin	DNA metabolism	mg/L	0.12/0.12	0.03/0.06	0.06/0.06	0.008/0.008
Fosfomycins	Fosfomycin	cell wall	mg/L	>1024/>1024	24/16	46/46	32/32
Lipopeptides/Polymixins	Colistin	cell membrane	mg/L	1/1.5	0.38/0.5	0.75/0.75	0.094/0.064
Tetracyclines	Tetracycline	protein synthesis	mg/L	16/16	8/8	8/16	4/4

/, results of two independent experiments; >, more resistant than the highest concentration tested.

[a ,b] See Table 1.

Supplementary Table S2 Transcripts with increased abundance in PA14Δ*hfq* versus PA14.

Supplementary Table S3 Transcripts with decreased abundance in PA14Δ*hfq* versus PA14.

1.2 Supplementary Figures

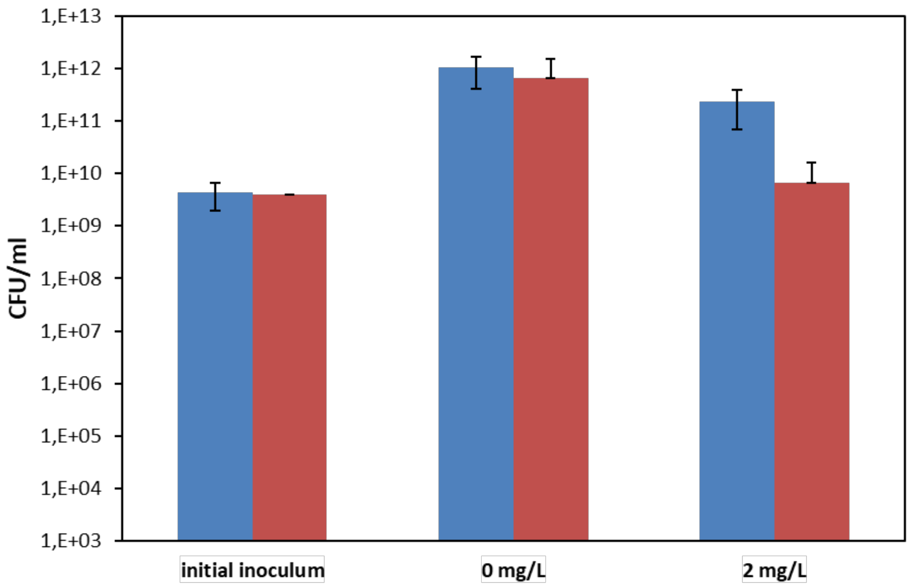

Supplementary Figure S1 Viability of early stationary phase cells of PA14 and PA14Δ*hfq* after treatment with gentamicin. Determination of the CFUs of PA14 (blue bars) and PA14Δ*hfq* (red bars) at an OD_{600} of 2.0 (initial inoculum) and in the absence (0 mg/L) or presence (2 mg/L) of gentamicin after 18 h of static growth in SCFM.

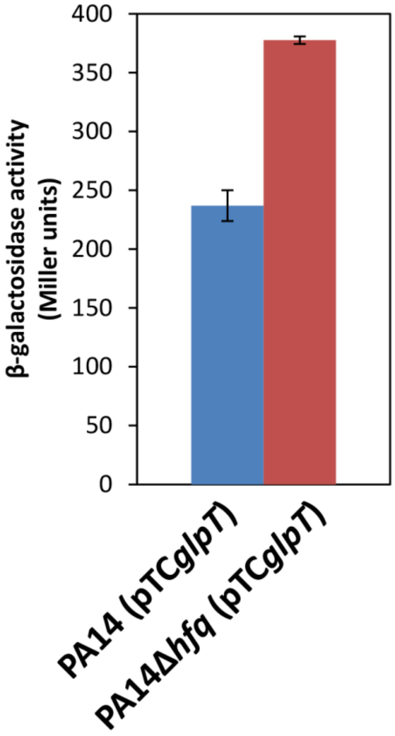

Supplementary Figure S2 The transcription of *glpT* is increased in the absence of Hfq. The strains PA14 (pTC*glpT*) (blue bar) and PA14Δ*hfq* (pTC*glpT*) (red bar) were grown in SCFM. Samples were withdrawn at an OD_{600} of 2.0. The bars represent the β-galactosidase values conferred by the plasmid pTC*glpT* encoded transcriptional *glpR-lacZ* fusion gene in the presence or absence of *hfq*. The error bars represent standard deviations from three independent experiments.

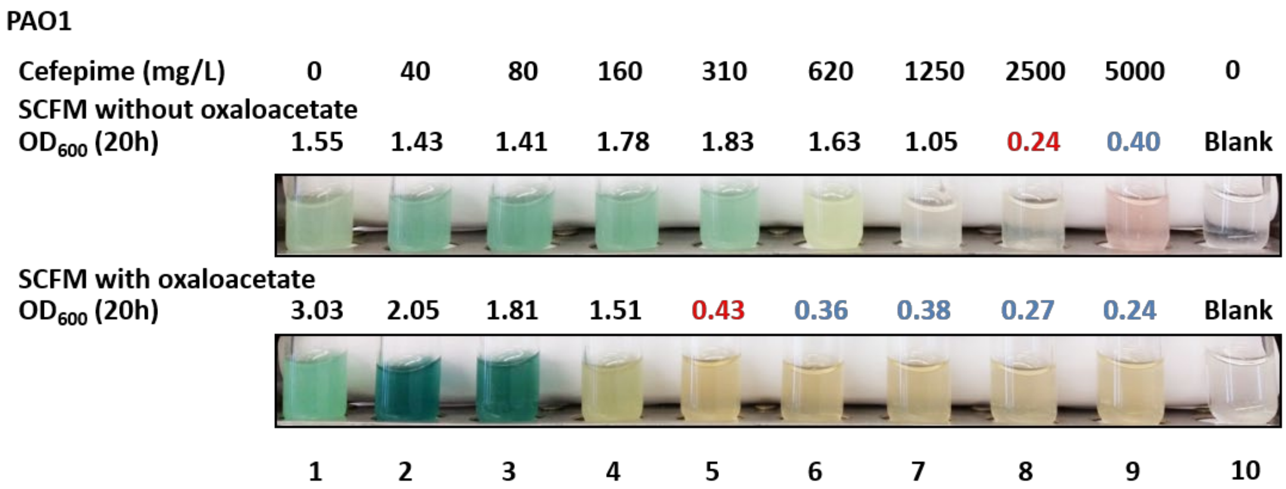

Supplementary Figure S3 Addition of OAA to SCFM results in increased sensitivity of PAO1 towards cefepime. The PAO1 culture was diluted to an initial OD_{600} of 0.5 and incubated for 20 h in SCFM with or without 40 mM OAA in the presence of different cefepime concentrations as indicated on top. Pictures were taken and the OD_{600} was measured 20 h after inoculation. The antibiotic concentrations in the presence of which the cells did not grow above OD_{600} of 0.5 (marked in red) were considered as MIC. All OD_{600} values obtained above this cefepime concentration are depicted in blue indicating toxicity. The experiments were performed in duplicate, revealing the same MICs. Only one representative experiment is shown.
